# Burnout and Stress Measurement in Police Officers: Literature Review and a Study With the Operational Police Stress Questionnaire

**DOI:** 10.3389/fpsyg.2020.00587

**Published:** 2020-05-07

**Authors:** Cristina Queirós, Fernando Passos, Ana Bártolo, António José Marques, Carlos Fernandes da Silva, Anabela Pereira

**Affiliations:** ^1^Faculty of Psychology and Education Sciences, University of Porto, Porto, Portugal; ^2^Psychology Unit of the Portuguese National Police, Lisbon, Portugal; ^3^Center for Health Technology and Services Research (CINTESIS), Department of Education and Psychology, University of Aveiro, Aveiro, Portugal; ^4^School of Health of the Polytechnic of Porto, Porto, Portugal; ^5^Department of Education and Psychology, University of Aveiro, Aveiro, Portugal

**Keywords:** burnout, distress, operational stress, police officers, questionnaire validation

## Abstract

Research has demonstrated that policing is a stressful occupation and that this stress has a negative impact on police officers’ mental and physical health, performance, and interactions with citizens. Mental health at the workplace has become a concern due to the costs of depression, anxiety, burnout, and even suicide, which is high among police officers. To ameliorate occupational health, it is therefore crucial to identify stress and burnout levels on a regular basis. However, the instruments frequently used to measure stress have not valorized the specificity of policing tasks. This study aims to: (i) conduct a literature review to identify questionnaires used to assess occupational stress and burnout among police officers; (ii) analyze the psychometric characteristics of a Portuguese version of Operational Police Stress Questionnaire (PSQ-Op); and, using the PSQ-Op and other questionnaires, (iii) to identify operational stress, burnout, and distress levels among Portuguese police officers. The literature review identified 108 studies which use a multiplicity of questionnaires to measure burnout or occupational stress among police officers, but few studies use specific police stress questionnaires. Sample sizes were mostly below 500 participants and studies were mainly developed in the last decade in the USA and Brazil, but also in another 24 countries, showing the extent of the interest in this topic. This study applied to 2057 police officers from the National Portuguese Police, a force policing urban centers, and used the PSQ-Op, as well the Spanish Burnout Inventory and the Kessler Psychological Distress Scale. The results show that the psychometric properties of the Portuguese version of PSQ-Op are adequate. Factorial analysis revealed two dimensions defined as social and work issues, which were associated with measures of distress and burnout. Fit indices suggested a second-order solution called operational police stress. Overall, and considering the scale range of each questionnaire, the results showed moderate values of operational stress, distress, and burnout. However, considering their cut-off points, 85% of the sample presented high operational stress levels, 11% critical values for burnout, and 28% high distress levels, with 55% of the sample at risk of a psychological disorder. These results reinforce the need to prevent stress and to invest in police officers’ occupational health.

## Introduction

According to recent systematic reviews, being a police officer seems to be a highly demanding and stressful occupation, due to the current characteristics of modern societies. For a police officer, those characteristics include: the uncertainty and danger related to the permanent threat of terrorist attacks, the increase of violence with firearms in urban areas, low human and material resources, team or supervision difficulties, criticism from citizens and society, and lack of understanding from family or friends (Cumming et al., 1965; Webster, 2013; Magnavita et al., 2018; Purba and Demou, 2019). Numerous studies have tried to map police officers’ stress and its sources, a topic highlighted in the 1980s by the NIOSH technical report (Hurrell et al., 1984), and in the 1990s by Norvell et al. (1993), whose study focused on the influence of gender differences on law enforcement officers. Brown and Campbell (1994), Violanti and Aron (1995), and Stinchcomb (2004) also studied the sources of policing stress. However, this topic has attracted more interest in the last decade, with studies developed, for example, by Hickman et al. (2011), Luceño-Moreno et al. (2016), and Violanti et al. (2017), all of whom continue to identify police officers’ stress sources and its negative impact on police officers’ health and job performance. More recently, Baldwin et al. (2019), Wassermann et al. (2019), and Ermasova et al. (2020) have contributed to the study of police officers’ stress and psychological/physical health. Related studies have focused more specifically on occupational stress (e.g., Agolla, 2009; Maran et al., 2015; Gutshall et al., 2017; Johnson et al., 2019), while others have investigated police officers’ burnout (e.g., Aguayo et al., 2017; Adams and Mastracci, 2019).

This has led to an increasing interest in police officers’ psychological well-being, with researchers emphasizing the negative impact of working with negative social situations, such as crime and death (Henry, 2004), which can affect mental health and elicit physical fatigue, compassion fatigue, and even moral suffering (Basinska and Wiciak, 2012; Papazoglou, 2016; Papazoglou et al., 2017, 2020; Violanti et al., 2019). Moreover, studies have concluded that job stress has consistently increased among police officers in the last decade, and this chronic job stress negatively affects both the person and the organization. Individually, it leads to poor mental health (Baldwin et al., 2019; Castro et al., 2019), work-family conflict (Griffin and Sun, 2018), non-adaptive coping strategies and job stress (LeBlanc et al., 2008; Zulkafaly et al., 2017), emotional labor (van Gelderen et al., 2007), burnout (Pines and Keinan, 2005, 2007; Rosa et al., 2015), and even suicide (Violanti, 1996; Blazina, 2017; Costa et al., 2019; Grassi et al., 2018). Organizationally, it affects performance (Shane, 2010; Bertilsson et al., 2019; Kelley et al., 2019), counterproductive work behaviors (Smoktunowicz et al., 2015), and inappropriate interactions with citizens, such as the use of excessive force (Neely and Cleveland, 2011; Mastracci and Adams, 2019).

A number of news sources have recently reported that France^1^ faces an increasing number of police officers committing suicide, especially after the intense work due to the “yellow vests/jackets” manifestations, while Spain^2^ and Portugal^3^ have also experienced several suicides of police officers, which motivated police officers to demonstrate in the streets and show their anger with job conditions in France^4^ and Portugal^5^. Hard working conditions and colleagues’ suicides elicit continuous suffering and psychological pain that affects police officers, their families, and their tasks in important domains of urban life: safety and security. Additionally, stressful situations can increase the use of antidepressants, anxiolytics, or tranquilizers to alleviate psychological suffering, with Portugal being one of the countries where this increased use is the highest in Europe (OECD, 2019), suggesting the need to invest in stress and anxiety prevention and in occupational health.

Despite the increased number of studies analyzing occupational stress and burnout among police officers, researchers frequently use measurement instruments developed for other professional groups which do not apply to the specificities of police tasks, including emotional labor and physical risks. This study aims to: (i) conduct a literature review to identify questionnaires that have been used to assess occupational stress and burnout among police officers; (ii) analyze the psychometric characteristics of a Portuguese version of Operational Police Stress Questionnaire (PSQ-Op), developed by McCreary and Thompson (2006), to assess the specificities of job stress among police officers; and, using the PSQ-op and other questionnaires, (iii) identify operational stress, burnout, and distress levels among Portuguese police officers.

Regarding burnout and occupational stress measurement among police officers, in the 1970s Freudenberger (1974) and Maslach (1976) identified the symptoms of burnout and defined burnout syndrome as a psychological disorder triggered by chronic exposure to work stress. Burnout has attracted considerable interest in the scientific community and has become a concern for workers, being recognized as a serious professional hazard and a psychosocial risk at work. The definition presented by Maslach and Jackson (1981) seems to be the most consensual, and states that burnout is a three-dimensional syndrome that affects workers whose job tasks are mainly related to helping and delivering care or services to other persons. Burnout is expressed by emotional exhaustion (feeling fatigued and powerless to provide more support to others), depersonalization (showing a disengaged, cynical, cold, and unsympathetic attitude toward persons at work, especially those who seek help or ask for services), and feelings of low professional achievement (feeling personal and professional inadequacy, and having a higher likelihood of committing errors during job tasks). Later, as a result of continuous research on burnout (Maslach and Leiter, 2016, 2017; Maslach, 2017) stated that burnout occurs more frequently among professionals who work with other persons, especially as service providers where, over the years, they must respond to the client’s demands in a society increasingly based on service exchanges, which elicits job stress.

Burnout appears as a response to chronic job stress (Schaufeli, 2017) and has become an epidemic phenomenon with costs for workers and organizations, which is a concern that has been repeatedly highlighted by the European Agency for Safety and Health at Work (EU-OSHA, 2018), namely with its “Healthy Workplaces” campaign. Moreover, several key organizations have reinforced the importance of burnout in modern society. On 10 October 2017, the World Health Organization (WHO) defined mental health in the workplace as the theme for World Mental Health Day, highlighting job stress among specific professional groups, and in 2019 the WHO defined suicide prevention as the theme^6^, alerting the public to the risk of suicide among specific professional groups. In September 2018, the European Foundation for the Improvement of Living and Working Conditions (EUROFOUND, 2018) published the report “Burnout in the workplace: A review of data and policy responses in the EU,” which found that burnout had become a serious problem in Europe and that measures were needed to assess its levels among different occupations. In May 2019, the WHO^7^ recognized burnout as an occupational phenomenon to be included in the next version of the International Classification of Diseases. Also in 2019, the European Agency for Safety and Health at Work (EU-OSHA, 2019a, b) referred again to “The value of occupational safety and health and the societal costs of work-related injuries and diseases.” Again in 2019, the results of the “Third European Survey of Enterprises on New and Emerging Risks (ESENER-3”) reinforced the negative impact of job stress and the importance of occupation health in preventing occupational stress among other psychosocial risks, a topic that the WHO^8^ also highlighted.

According to Lazaus and Folkman (1984, p. 21), “psychological stress, therefore, is a relationship between the person and the environment that is appraised by the person as taxing or exceeding his or her resources and endangering his or her well-being.” Based on this definition, the concept of stress at the workplace, job stress, or occupational stress can be defined as a “pattern of physiological, emotional, cognitive, and behavioral responses that occur when workers are presented with work demands not matched to their knowledge, skills, or abilities and which challenge their ability to cope” (Patel et al., 2017, p. 1), negatively influencing the worker’s wellbeing, performance, and productivity (Quick and Henderson, 2016). Moreover, stress, especially job stress and occupational stress, are related and can predict burnout, since job stress can result from the relationship between job demands and job resources, or from the effort-reward imbalance (Peiró et al., 2001; Lin et al., 2013; Chirico, 2016; Patel et al., 2017; Salvagioni et al., 2017; Wang et al., 2017). Furthermore, burnout can be a long-term process of resource depletion and inadequate responses to chronic job stress (Maslach et al., 2001; Schaufeli, 2017). Burnout is difficult to distinguish from depression since they share similar symptoms (Bianchi et al., 2015; Golonka et al., 2019; Koutsimani et al., 2019; Bianchi, 2020).

Using instruments that allow burnout and stress to be measured is therefore a vital necessity before designing intervention programs for resilience, stress management, and burnout or suicide prevention. However, for police officers as a professional group, those instruments must be chosen carefully, considering the specificity of their policing tasks. To identify the instruments used to measure burnout and stress among police officers, a literature search was performed between January and December 2019 on the EBSCO database of scientific papers, using the following search expression: “police officers” and “burnout or stress” and “instruments or tools or scale or questionnaire or inventory or measurement or assessment or evaluation.” The search found 191 scientific published papers after removing duplicated references. However, 49 papers were focused exclusively on post-traumatic stress disorder; 26 were written in languages other than English, Portuguese, or Spanish, or the complete paper was unavailable; 5 were theoretical papers; and 3 used qualitative methods. Thus, a final number of 108 studies were analyzed, identifying the publication year, number of participants, country of the sample, and instruments used for burnout and stress or occupational stress measurement.

**FIGURE 1:**
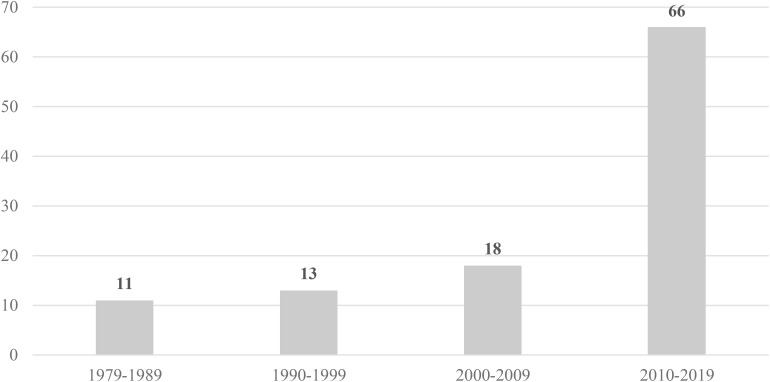
Distribution of papers according year of publication.

**FIGURE 2:**
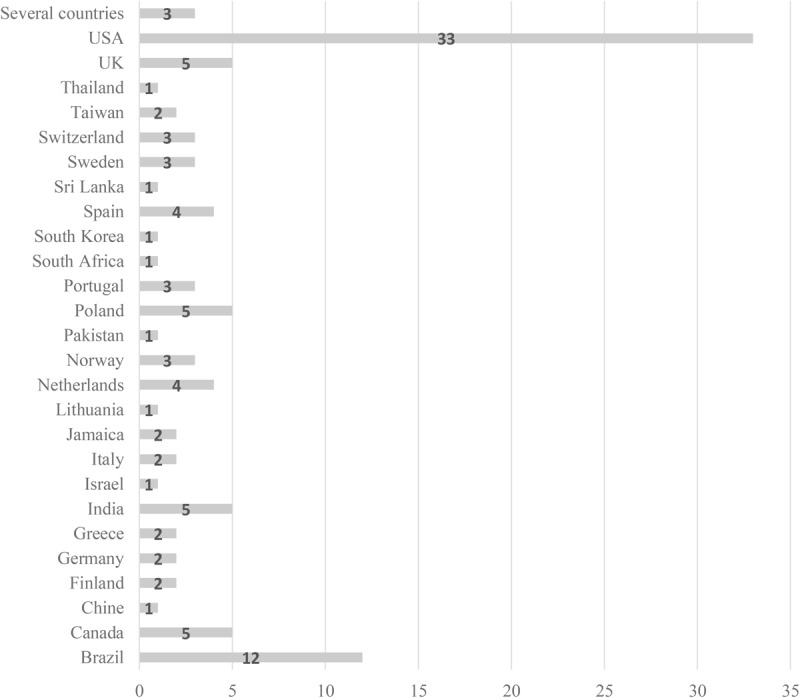
Distribution of papers according country of the study.

Results of the literature review (Table 1) revealed that most of the studies are recent (Figure 1), though the interest in questionnaires to assess burnout or job stress began in the 1970s. In detail, 11 studies were published between 1979 and 1989, 13 between 1990 and 1999, 18 between 2000 and 2009, and 66 between 2010 and 2019. The samples came from 26 countries (Figure 2), mostly the USA (33), but Brazil appears with 12 studies, 4 or 5 studies were found in the UK, Poland, India, Canada, Spain, and the Netherlands, and 2 or 3 in Switzerland, Sweden, Portugal, Taiwan, Jamaica, Italy, Greece, Germany, and Finland. Three papers used samples from several countries in the same study. Finally, countries with only one study included Thailand, Sri Lanka, South Korea, South Africa, Pakistan, Lithuania, Israel, and China. These data express the global interest of scientific research in stress among police officers.

**TABLE 1:** Studies using questionnaires to measure burnout or occupational stress of police officers.

**References**	**Year**	**Country**	**Sample (N)**	**Burnout measure**	**Stress measure**
Adams and Mastracci (2019)	2019	USA	271	Maslach Burnout Inventory (MBI; Maslach et al.)	
Alberti et al. (2016)	2016	Spain	74	MBI	
Albuerne et al. (2015)	2015	Spain	462		Social Work Stress Appreciation Scale
Almeida et al. (2018)	2018	Brazil	519		Escala de Stresse no Trabalho (Paschoal and Tamayo)
Anshel and Brinthaupt (2014)	2014	USA	11		Perceived Stress Scale (PSS; Cohen et al.)
Anson et al. (1997)	1997	USA	48		Personalized Assessment Stress Scale (PASS; Morse and Frost)
Antoniou (2009)	2009	Greece	512		Antoniou Police Stress Inventory (Karanika-Murray et al.)
Arnetz et al. (2013)	2013	Sweden	75		Bodily Symptom Scale (Petterson et al.) Exaustion Questionnaire (Appels et al.)
Baka (2015)	2015	Poland	625	Oldenburg Burnout Inventory (OLBI; Demerouti et al.)	
Bakker and Heuven (2006)	2006	Netherlands	101	MBI	
Basinska and Wiciak (2012)	2012	Poland	89	OLBI	
Basinska and Daderman (2019)	2019	Poland	234	OLBI	
Basinska et al. (2014)	2014	Poland	169	OLBI	
Bergman et al. (2016)	2016	USA	47		Police Stress Questionnaire (McCreary and Thompson)
Brown and Cooper (1996)	1996	UK	500		Occupational Stress Inventory (OSI)
Brown and Fielding (1993)	1993	UK	489		Occupational stress inventory (Davidson and Cooper)
Burke (1993)	1993	Canada	828	MBI	Job-Related Stress
Burke (1994)	1994	Canada	828	MBI	
Burke and Deszca (1986)	1986	Canada	828	MBI	Sources of Experienced Stress Stressful Life Events
Burke and Mikkelsen (2005)	2005	Norway	766	MBI	
Burke and Mikkelsen (2006)	2006	Norway	766	MBI	
Burke et al. (1984)	1984	Canada	426	MBI	Sources of Experienced Stress Stressful Life Events
Carvalho et al. (2008)	2008	Brazil	394		PSS Stress Symptoms Inventory (SSI)
Charles et al. (2011)	2011	USA	430		PSS
Chen (2009)	2009	Taiwan	156		Job stress scale for intra-organization and extra-organization factors
Chitra and Karunanidhi (2018)	2018	India	250		Occupational Stress Inventory (OSI)
Chongruksa et al. (2012)	2012	Thailand	42		Symptoms Checklist 90 (SCL-90)
Collins and Gibbs (2003)	2003	UK	1206		General Health Questionnaire (GHQ)
Couto et al. (2012)	2012	Brazil	327		Lipp Stress Symptoms Inventory (LSSI, Lipp)
Daderman and Colli (2014)	2014	Sweden	101		Coping Resources Inventory (CRI)
Ellrich (2016)	2016	Germany	1742	MBI	
Euwema et al. (2004)	2004	Netherlands	358	MBI	
Figueiredo-Ferraz et al. (2014)	2014	Portugal	245	Spanish Burnout Inventory (Gil- Monte)	
Garbarino and Magnavita (2019)	2019	India	852	Burnout Inventory (BI-MK, Misra)	Occupational Stress Index (OSI)
Garbarino et al. (2013)	2013	Italy	289		Demand/control/support (DCS; Karasek) Effort/reward imbalance (ERI; Siegrist)
Gerber et al. (2010a)	2010	Switzerland	460		Trier Inventory for the Assessment of Chronic Stress (TICS; Schulz et al.)
Gerber et al. (2010b)	2010	Switzerland	533		Trier Inventory for the Assessment of Chronic Stress (TICS; Schulz et al.)
Gershon et al. (2009)	2009	USA	1072		Police Stress Scale and Police Coping Scale (Beehr et al.)
Gomes and Afonso (2016)	2016	Portugal	95		Global Level of Stress (Kyriacou)
Goodman (1990)	1990	USA	199	Staff Burnout Scale for Police and Security Officers (SBS-PS; Jones)	Police Officer History Questionnaire (Goodman)
Griffin and Sun (2018)	2018	USA	138	MBI	PSS
Gutshall et al. (2017)	2017	USA	32	MBI	PSS
Hartley et al. (2013)	2013	USA	452		PSS Spielberger Police Stress Survey (Spielberger et al.)
Hassell et al. (2011)	2011	USA	87		Stress assessed by five items
Hawkins (2001)	2001	USA	452	MBI	
Houdmont (2013)	2013	UK	139	MBI	
Hu et al. (2017)	2017	Chine	273	MBI	Questionnaire on the Experience and Evaluation of Work (Hu et al.)
Husain et al. (2014)	2014	Pakistan	315		Depression Anxiety and Stress Scale (Lovibond and Lovibond)
Kaplan et al. (2017)	2017	USA	72		PSS Police Stress Questionnaire (McCreary and Thompson)
Kirkcaldy (1993)	1993	USA, Spain, Germany, UK, Ireland, Holland, Finland, Denmark	42		Occupational Stress Indicator (Cooper et al.)
Kirkcaldy and Cooper (1992)	1992	Germany North Ireland	156		Occupational Stress Indicator (Cooper et al.)
Kirkcaldy et al. (1994)	1994	Germany North Ireland	156		Occupational Stress Indicator (Cooper et al.)
Kirkcaldy et al. (1995)	1995	UK	533		Occupational Stress Indicator (Cooper et al.)
Kop and Euwema (2001)	2001	Netherlands	358	MBI	
Kop et al. (1999)	1999	Netherlands	358	MBI	
Korre et al. (2014)	2014	USA	951		Daily perceived stress level
Kuo (2014)	2014	Taiwan	1315		Identification of job stressors
Kwak et al. (2018)	2018	South Korea	466	MBI	
Lambert et al. (2017)	2017	India	827	Burnout questions (adapted from Wright and Saylor)	Job stress (Crank et al)
Lambert et al. (2019)	2019	India	1000	Wright and Salyor burnout measures	
Lester (1982a)	1982	USA	73		Subjective level of stress from job conditions
Lester (1982b)	1982	USA	41		Self-evaluation of stress questionnaire (Willcher)
Lester and Mink (1979)	1979	USA	15		Identification of job stressors
Lester and Solis (1980)	1980	USA	20		Self-evaluation of stress questionnaire (Willcher)
Lester et al. (1984)	1984	USA	55		Stress Profile (Girdano and Everly)
Lester et al. (1985)	1985	USA	48		Eight stress tests (Girdano and Everly)
Levitov and Thompson (1981)	1981	USA	250		Self Report Form (Cattell)
Lima et al. (2018)	2018	Brazil	80	Burnout Questionnaire (based on MBI; Jbeili)	
Lipp (2009)	2019	Brazil	418		Lipp Stress Symptoms Inventory (LSSI, Lipp) Police Officers Stressors Questionnaire (POSQ)
Lipp et al. (2017)	2017	Brazil	1837		Lipp Stress Symptoms Inventory (LSSI, Lipp) Job Stressor Sources Inventory (IFET)
Louw (2014)	2014	South Africa	505	Shirom–Melamed burnout measure (SMBM)	
Lucas et al. (2012)	2012	USA	115		Police Stress Survey (Spielberger et al.)
Maran et al. (2015)	2015	Italy	617		Police Stress Questionnaire Distress Thermometer
Maria et al. (2019)	2019	Germany	811	Copenhagen Burnout Inventory (CBI; Kristensen et al.)	
Martinussen et al. (2007)	2007	Norway	223	MBI	
McCarty et al. (2007)	2007	USA	1100		Questions about stress symptoms and stress sources
McCarty et al. (2019)	2019	USA	13146	MBI	Law Enforcement Organizational Survey C (LEO C)
Nelson and Smith (2016)	2016	Jamaica	134		Well-being Process Questionnaire (WPQ)
Padyab et al. (2016)	2016	Sweden	1554	MBI	Stress of Conscience Questionnaire (SCQ)
Papazoglou et al. (2018)	2018	Finland	1173	Compassion Fatigue Test; Compassion Satisfaction and Fatigue Self-Test for Helpers (CSF)	
Patterson (1992)	1992	USA	4500		Spielberger’s Police Stress Survey
Patterson (2003)	2003	USA	233		Police stress questions (based on Spielberger’s Police Stress Survey) Psychological distress (based on CES-D; Radloff)
Pelegrini et al. (2018)	2018	Brazil	84		Job Stress Scale
Perez et al. (2010)	2010	USA	28	MBI	
Pines and Keinan (2007)	2007	Israel	1010	Burnout Measure Short (BMS, Pines)	Self-Report Questionnaire of Stressors
Queirós et al. (2013)	2013	Portugal	274	MBI	
Romosiou et al. (2018)	2018	Greece	77		PSS
Roz and Raval (2017)	2017	India	852	Burnout Inventory (BI-MK; Misra)	Occupational Stress Index (OSI)
Russell et al. (2014)	2014	USA	482	MBI	Stress (Spielberger et al.)
Santana et al. (2012)	2012	Brazil	53		Lipp Stress Symptoms Inventory (LSSI, Lipp)
Sarason et al. (1979)	1979	USA	18		Inventory of Hostility (Endler and Hunt)
Schaible and Gecas (2010)	2010	USA	109	MBI	Emotional Labor Scale (Best et al.); Emotional Work Requirements Scale Community-oriented policing dissonance subscales
Schilling et al. (2019)	2019	Switzerland	201	Shirom–Melamed Burnout Measure (SMBM)	PSS
Schlichting et al. (2014)	2014	Brazil	1069		Occupational Stress Indicators (OSI)
Shipley and Baranski (2002)	2002	Canada	54		
Silveira et al. (2005)	2005	Brazil	60	MBI	
Smoktunowicz et al. (2015)	2015	Poland	625	Oldenburg Burnout Inventory (OLBI; Demerouti et al.)	Quantitative Workload Inventory (Spector and Jex) Psychosocial Working Conditions Questionnaire (Widerszal-Bazyl and Cieslak)
Solana et al. (2013)	2013	Spain	747	MBI	
Summerlin et al. (2010)	2010	USA	787		Police Stress Questionnaire (PSQ-Op and PSQ-Org)
Talavera-Velasco et al. (2018)	2018	Spain	223	MBI	DECORE-21 (Talavera)
Tang and Hammontree (1992)	1992	USA	60		Police Stress Survey (Spielberger et al.)
Tavares and Lautert (2017)	2017	Brazil	416		Questions about stress and cortisol
Trombka et al. (2018)	2018	Brazil	16	MBI BCSQ-12 Burnout Clinical Subtype Questionnaire (Montero-Marin et al.)	PSQ Police Stress Questionnaire
Vuorensyrja and Malkia (2011)	2011	Finland	2821	Bergen Burnout Indicator (BBI-15)	Police Personnel Barometer (PPB)
White et al. (1985)	1985	USA	355	MBI	Police Stress Inventory (Spielberger et al.)
Wickramasinghe and Wijesinghe (2018)	2018	Sri Lanka	750	Burnout Clinical Subtype Questionnaire (BCSQ-36)	
Wray and Jarrett (2019)	2019	Jamaica	305	MBI	
Zukauskas et al. (2009)	2009	Lithuania	314		Specific questions for job stress in Lithuania

**FIGURE 3:**
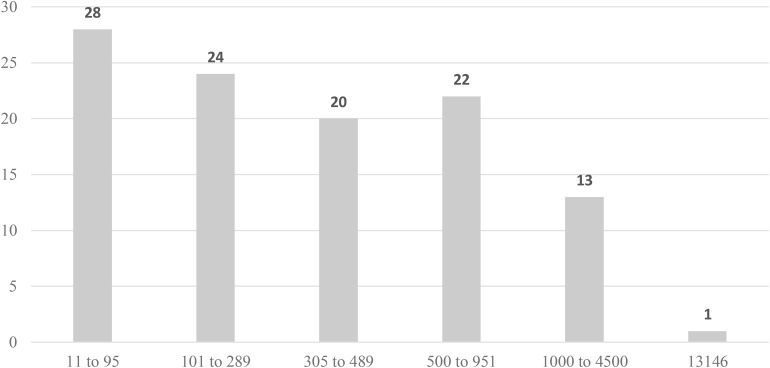
Distribution of papers according sample size.

The sample sizes varied between 11 and 13,146 participants (*M* = 595; *SD* = 1358.56). However, a more detailed analysis (Figure 3) revealed that 28 studies sampled 11–95 participants, 24 studies sampled 101–289, 20 studies sampled 305–489, 22 studies sampled 500–951, and 13 studies sampled 1000–4500 participants. One study collected data from 13,146 police officers in the USA (McCarty et al., 2019).

**FIGURE 4:**
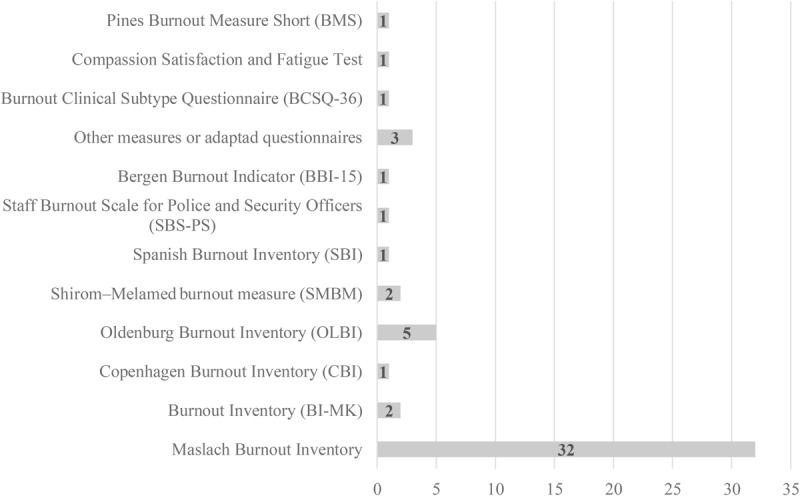
Distribution of papers according burnout measurement instrument.

**FIGURE 5:**
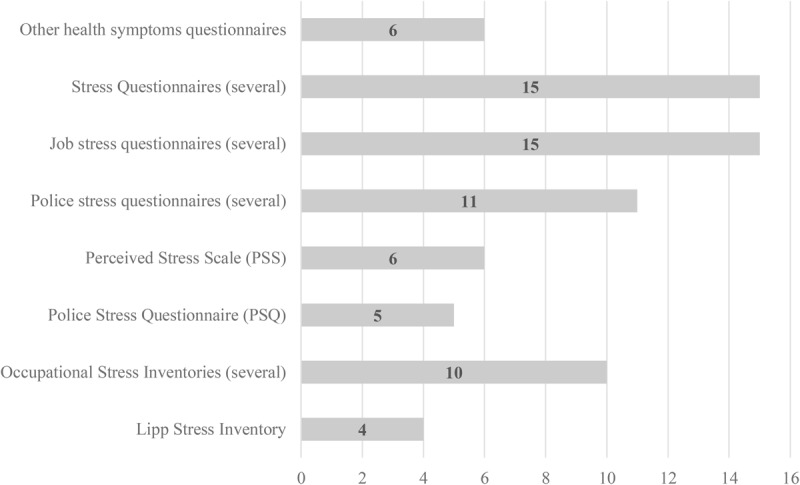
Distribution of papers according stress measurement instrument.

The analysis of measurement instruments revealed that 51 studies measured burnout (Figure 4), with the Maslach Burnout Inventory being prevalent (32 studies), while the Oldenburg Burnout Inventory was used in five studies. Three studies used other measures or developed questionnaires adapted from other instruments, while nine studies used specific but different burnout measures. Measures of job stress were found (Figure 5) in 72 studies: six used the Perceived Stress Scale, four used the Lipp Stress Inventory (from Brazil), five used the Police Stress Questionnaire, and 11 used several different police stress questionnaires. However, 10 studies used several occupational stress inventories, 15 used several job stress questionnaires, 15 used several stress questionnaires, and six used other instruments assessing health symptoms other than stress. This review revealed the proliferation of stress measures, although some studies already used specific police stress questionnaires. It can be concluded that measuring burnout and stress among police officers is a concern for the scientific community.

In Portugal, for burnout measurement among police officers, a study used the Spanish Burnout Inventory (Gil-Monte, 2011) and demonstrated that this questionnaire had adequate psychometric properties for a sample of Portuguese police officers (Figueiredo-Ferraz et al., 2014). Another study (Queirós et al., 2013) used the Maslach Burnout Inventory, analyzing only Cronbach’s alphas but not validating a Portuguese version. Regarding stress measurement, one study used a global measure of stress (Gomes and Afonso, 2016), but no studies were found with specific stress measures for policing. Following the analysis of the specific stress instruments found in the literature review, we decided to translate and validate a Portuguese version of the Police Stress Questionnaire for operational stress. The Police Stress Questionnaire (McCreary and Thompson, 2006) is a short measure (20 items) allowing for the assessment of operational or organizational police stress, it is freely available for research purposes and has established stress levels with cut-off points. Since some studies used global measures of stress or stress symptoms, it was decided to also use the short questionnaire Kessler Psychological Distress Scale (K10), which has a recent Portuguese version (Pereira et al., 2019). Thus, this study can contribute to the development of a Portuguese version of a specific police stress instrument, and to identify stress and burnout levels of a sample of police officers using validated instruments.

## Materials And Methods

### Participants

The sample was composed of 2057 police officers of the Portuguese National Police (*Polícia de Segurança Pública, PSP*), a police force that works only in the cities of all 18 Portuguese districts and the Azores and Madeira Islands. The sample constitutes nearly 10% of this force and all districts were represented: Lisbon contributed 45% of the sample, Porto 19%, Setubal 6%, Faro and Azores 4%, Madeira 3%, and other districts between 0.3 and 2.5%.

Regarding police officers’ positions, 78.8% were in the “*agent*” category (the lowest-ranking officer), 14.6% were “chief,” and 6.5% commander (the highest rank). The most frequent tasks were patrolling (52%), criminal investigation (17%), and road traffic management (13%). Other participants worked in integrated special police units, rapid intervention teams, specific proximity teams (e.g., schools or elderly safety programs), administrative services, and commander teams.

The age of the participants varied between 21 and 65 years old (*M* = 42.47; *SD* = 8.785), with 33.4% between 21 and 38 years, 32.5% between 39 and 45, and the rest between 46 and 65. Job experience in the Portuguese National Police varied between 1 and 41 years (*M* = 19.267; *SD* = 9.036), with 32.3% of the sample between 1 and 14 years, 34.7% between 15 and 23 years, and the rest between 24 and 41 years. Regarding gender, 92% were men and 8% women, while overall women represent nearly 10% of the police force. To avoid the possible identification of individuals from the matching of position, age, gender, and district, no statistical analyses were performed that combined these data, and no other sociodemographic data were collected.

### Measures

The questionnaire was composed of four major groups of questions, the first characterizing the sociodemographic data (age, sex, job experience, district, position, and job task). The second group was composed of the Operational Police Stress Questionnaire (PSQ-Op), developed by McCreary and Thompson (2004, 2006) to assess the specificities of job stress among police officers both for operational and organizational stress sources (PSQ-Op and PSQ-Org). This study used the operational stress sources only. The PSQ-Op questionnaire is composed of 20 items evaluated on a 7-point scale ranging from 1 (“not at all stressful” or “no stress at all”) to 7 (“very stressful” or “a lot of stress”), with 4 indicating moderate stress. The authors were contacted by email to obtain permission for the Portuguese version, but no answer was obtained for the PSQ-Op as it is provided free for non-commercial, educational, and research purposes^9^. In later developments, McCreary et al. (2017) established norms and cut-off values, with values below 2.0 indicating low stress, between 2.1 and 3.4 moderate stress, and above 3.5 high stress. As far as we know, no Portuguese version of the PSQ-Op has been published, and two psychologists (one conducting research about policing and police forces, another working with police officers) translated the questionnaire into Portuguese. Another researcher, unfamiliar with police officers’ work, subsequently back-translated the questionnaire into English and compared it with the original version. Finally, these three researchers discussed each item with two police officers (a patrol police officer and a police station commander) until a lexical and cultural consensus was obtained, including suggestions from the police officers to add some examples adapted for Portuguese situations (Table 2). A pilot study was performed with 20 police officers to ensure that the questionnaire was easy to complete and was applicable to the Portuguese situation, and no major changes were made.

**TABLE 2:** PSQ-Op original and Portuguese versions.

**Original PSP-Op (McCreary and Thompson (2006))**			**Portuguese version of PSQ-Op**		
**No stress at all**	**Moderate stress**	**A lot of stress**	**Nenhum stress**	**Stress moderado**	**Muito stress**
**1**	**4**	**7**	**1**	**4**	**7**
1. Shift work			1. Trabalhar por turnos		
2. Working alone at night			2. Trabalhar sozinho à noite		
3. Over-time demands			3. Exigências relacionadas com horas extra ou serviço imprevisto		
4. Risk of being injured on the job			4. Risco ou possibilidade de ser ferido durante o trabalho		
5. Work related activities on days off (e.g., court, community events)			5. Ter atividades relacionadas com o trabalho em dias de folga (ex.: comparecer em tribunal, eventos na comunidade)		
6. Traumatic events (e.g., MVA, domestics, death, injury)			6. Acontecimentos traumáticos (ex.: acidentes rodoviários, violência doméstica, mortes, agressões)		
7. Managing your social life outside of work			7. Gerir a sua vida social fora do trabalho		
8. Not enough time available to spend with friends and family			8. Ter pouco tempo disponível para passar com os amigos ou família		
9. Paperwork			9. Aspetos burocráticos do serviço (ex.: relatórios)		
10. Eating healthy at work			10. Conseguir comer de forma saudável no trabalho		
11. Finding time to stay in good physical condition			11. Conseguir arranjar tempo para ficar em boa forma física		
12. Fatigue (e.g., shift work, over-time)			12. Andar cansado (ex.: por trabalhar por turnos, horas extraordinárias)		
13. Occupation-related health issues (e.g., back pain)			13. Ter problemas de saúde relacionados com a profissão (ex.: dores de costas ou dores nas pernas por patrulhar a pé)		
14. Lack of understanding from family and friends about your work			14. Falta de compreensão da família e amigos em relação às exigências do seu trabalho		
15. Making friends outside the job			15. Conseguir fazer amigos fora do trabalho		
16. Upholding a “higher image” in public			16. Conseguir manter uma boa imagem na sociedade		
17. Negative comments from the public			17. Escutar comentários negativos por parte dos cidadãos		
18. Limitations to your social life (e.g., who your friends are, where you socialize)			18. Ter limitações na sua vida social (ex.: quem são os seus amigos, locais onde convive)		
19. Feeling like you are always on the job			19. Sentir-se como se estivesse sempre a trabalhar		
20. Friends/family feel the effects of the stigma associated with your job			20. Os amigos e família sentirem os efeitos do estigma associado à sua profissão		
The Operational Police Stress Questionnaire is provided free for non-commercial, educational, and research purposes. Cite as: - McCreary, D.R., and Thompson, M.M. (2013). The Operational Police Stress Questionnaire (PSQ-Op). Measurement Instrument Database for the Social Science. Retrieved from www.midss.ie			O Questionário de Stress Operacional é de acesso livre para efeitos de uso não comercial, educacional e investigação. Citar como: - versão original de: McCreary, D. R., and Thompson, M. M. (2013). The Operational Police Stress Questionnaire (PSQ-Op). Measurement Instrument Database for the Social Science. Retrieved from www.midss.ie		
			- Queirós, C., Passos, F., Bártolo, A., Marques, A. J., Silva, C. F., and Pereira, A. (2020). Burnout and stress measurement in police officers: literature review and a study with the operational police stress questionnaire. *Front. Psychol.* 11, 587. doi: 10.3389/fpsyg.2020.00587.		

The third group of questions was composed from the Spanish Burnout Inventory (SBI, Gil-Monte, 2011), using a Portuguese version already tested on police officers, having demonstrated adequate psychometric properties (Figueiredo-Ferraz et al., 2014). This instrument considers burnout as a process of cognitive and emotional deterioration, involving attitudes of indifference and guilt (Gil-Monte and Manzano-García, 2015). It includes 20 items organized on four scales: (1) enthusiasm for the job (demonstrating, for instance, the ambition to accomplish a person’s professional goals because they are a source of personal achievement); (2) psychological exhaustion (emotional and physical exhaustion related to job tasks, increased by dealing every day with people who present difficulties or problems); (3) indolence (negative attitudes of indifference and cynicism when dealing with persons demanding things related to a person’s job tasks); and (4) guilt (negative feelings, behaviors, and attitudes in the workplace, elicited by interactions during labor relations). Each item is assessed by a 5-point frequency scale ranging from 0 (never) to 4 (very frequent or every day). Low scores on Enthusiasm for the Job, along with high scores on Psychological Exhaustion, Indolence, and Guilt, indicate high levels of burnout. Scores for each of the four scales are calculated using the mean of the items that compose each scale, and a global score for burnout is then calculated after reversing the items of the Enthusiasm scale. According to Poletto et al. (2016), it is possible to use percentile analysis to identify burnout at very low levels (*P* ≤ 10), low levels (11 < *P* ≤ 33), moderate levels (34 < *P* ≤ 66), high levels (67 < *P* ≤ 89), and critical levels (*P* ≥ 90).

The fourth and last group of questions was composed from the Kessler Psychological Distress Scale (K10), from Kessler et al. (2002, 2003). We used the Portuguese version by Pereira et al. (2019), who described K10 as having 10 items that assess the frequency of non-specific psychological distress symptoms during the last month, being a self-report measure based on questions about the symptoms of anxiety and depression. All items are assessed on a 5-point scale (1 = “none of the time” to 5 = “all of the time”) and the sum of the scores indicates the stress level, where high scores correspond to high stress levels. Using this sum it is possible to identify cut-off points of stress levels, where 10–15 points correspond to “low distress,” 16–21 points to “moderate,” 22–29 points to “high,” and 30–50 points to “very high.” Values between 22 and 50 points indicate a risk of developing a psychological disorder (Andrews and Slade, 2001; Pereira et al., 2019).

### Procedure

After formal authorization by the Directorate of the Portuguese National Police to develop the study and collect data among the police officers, an online questionnaire was prepared on Google Forms with a link inviting participation in a study of burnout and occupational stress among police officers. The Directorate disseminated this link to the police officers using their professional email addresses. There was no direct contact between participants and researchers, and data were collected in September and October 2019. No exclusion criteria existed, and participation was voluntary. The participation rate was nearly 10% of the number of police officers that constitute this police force. Researchers were unable to identify how many police officers read the email and/or followed the link and decided not to participate. This study was carried out in accordance with the recommendations of the Ethics guidelines of the FPCEUP Ethics Committee, having online informed consent from all participants in accordance with the Declaration of Helsinki. Thus, before responding to the questionnaire, participants were asked to provide their informed consent, with the notification that data would be gathered anonymously. Data were accessed by one researcher only, who downloaded the Excel file and converted it to SPSS format.

### Data Analysis

Statistical analysis was performed using Statistical Package for Social Sciences, version 24 (SPSS Inc., Chicago) and Analysis of Moment Structures (AMOS) version 24. In the first stage, preliminary analyses were conducted in order to assess descriptive statistics, and normality and non-multicollinearity at item level. To test the factorial structure of the PSQ-Op, we used a combination of Exploratory Factor Analysis (EFA) and Confirmatory Factor Analysis (CFA). The sample was randomly split into two samples through the randomization function in SPSS. With the first part of the sample (*n* = 636), an EFA using Principal Axis Factoring (PAF) with direct oblimin rotation was conducted to identify a viable factor structure by extracting the minimum number of factors that explained the maximum variance in the 20-item scale. With the remaining sample, a CFA was performed to verify if the solution obtained from the EFA presented an acceptable fit. The root mean square error of approximation (RMSEA), the comparative fit index (CFI), and the standardized root mean square residual (SRMR) were the three indicators used to evaluate the model’s fit. An acceptable-fit model was determined if RMSEA ≤ 0.08, CFI > 0.90, and SRMR < 0.10 (Kline, 2005). The chi-square test (χ^2^) was reported for completeness, but not used to check the model fit due to its sensibility to large samples (Kelloway, 1995). Based on the multivariate normality violations, CFA used the maximum likelihood estimation with bootstrapping (1000 resamples). The Bollen-Stine bootstrap *p* was an index fit also reported. Alternative factor models were generated and tested according to the modification indexes (MI > 11).

Convergent validity was assessed by computing the average variance extracted with values of AVE ≥ 0.50 indicating satisfactory validity. In turn, in order to investigate the evidence of discriminant validity, we examined whether the AVE values were equal to or greater than the squared correlation between the factors (*r*^2^_DV_) (Maroco, 2014). Following the model specification, reliability was investigated using (a) Cronbach’s alpha coefficient and (b) composite reliability for each factor and for the overall scale.

Finally, the relationship between the PSQ-Op dimensions and distress and burnout symptoms was determined from the Pearson correlation coefficients including the entire sample, as well as the descriptive statistics, which allowed us to identify burnout, distress, and operational stress levels.

## Results

Since there are no specific measures for operational stress among police officers, it was necessary to evaluate the psychometric properties of the Portuguese version of the PSQ-Op before identifying burnout, distress, and operational stress levels.

### Preliminary Analysis: Item Properties

As shown in Table 3, all possible Likert-scale answer values for each item were observed. The mean for most items was close to 5. The overall mean response for the 20 items was 4.97 (*SD* = 0.45) No deviations from the normal distribution were found considering skewness (*Sk*, < 3.0) and kurtosis (*Ku*, ≤ 7.0) absolute values (Byrne, 2016). All items presented significant positive corrected item-total correlations (≥0.40) and low variation in reliability if the item was deleted. Inter-correlations among all items were significant and no multicollinearity was obtained (0.390 ≤ *r* ≤ 0.731) (Tabachnick and Fidell, 2001). Based on this analysis, 20 items were retained for subsequent analyses.

**TABLE 3:** Descriptive statistics about PSQ-Op items (*n* = 2057).

**Item**	**M**	**Min**	**Max**	**SD**	**Skewness**	**Kurtosis**	**Corrected item-total correlation**	**Cronbach’s alpha if item deleted**
1	5.19	1	7	1.764	−0.747	−0.330	0.664	0.955
2	5.36	1	7	1.863	−0.942	−0.231	0.631	0.956
3	5.46	1	7	1.648	−0.932	−0.031	0.630	0.955
4	5.08	1	7	1.809	−0.613	−0.673	0.562	0.955
5	5.53	1	7	1.681	−1.052	0.210	0.596	0.955
6	5.14	1	7	1.750	−0.739	−0.467	0.544	0.955
7	4.34	1	7	1.884	−0.199	−1.038	0.557	0.955
8	5.27	1	7	1.676	−0.763	−0.331	0.586	0.954
9	4.92	1	7	1.707	−0.548	−0.607	0.451	0.955
10	5.15	1	7	1.752	−0.749	−0.396	0.633	0.955
11	4.94	1	7	1.730	−0.567	−0.586	0.616	0.955
12	5.57	1	7	1.574	−1.025	0.260	0.681	0.954
13	5.21	1	7	1.740	−0.782	−0.363	0.571	0.954
14	4.56	1	7	1.861	−0.362	−0.928	0.561	0.955
15	4.02	1	7	1.995	−0.037	−1.207	0.650	0.955
16	4.13	1	7	1.992	−0.123	−1.175	0.630	0.955
17	5.21	1	7	1.758	−0.736	−0.551	0.545	0.955
18	4.50	1	7	1.915	−0.293	−1.063	0.722	0.954
19	5.03	1	7	1.792	−0.637	−0.647	0.670	0.954
20	4.76	1	7	1.837	−0.459	−0.847	0.660	0.954

### Exploratory Factor Analysis (EFA)

In order to examine the factor structure, an EFA was conducted based on a randomized split of the data in the sample (*n* = 636). EFA using principal axis factor analysis with promax rotation determined the factor structure of the 20 items of the questionnaire. The Kaiser-Meyer-Olkin (KMO) measure presented a value of 0.964 and Bartlett’s test of sphericity was significant (χ^2^ = 9621.92, *p* < 0.001), validating the correlation matrix structure. EFA yielded a 20-item measure with a two-factor solution (Table 4): nine items included content related to social issues (items 7, 8, and 14–20), which expressed the feeling that a police officer is always on the job, as well as facing difficulties in managing personal life or balancing work and family, and having to deal with the public/social image of the police force and citizens’ negative comments; the other eleven items included content that reflected work issues (items related to specific details of policing tasks such as shift work, paperwork, injuries, fatigue, and traumatic events). These two factors together (social issues and work issues) accounted for 60.30% of the total variance. A good internal consistency for each factor was estimated using Cronbach’s alpha coefficients: factor 1, α = 0.937 and factor 2, α = 0.933.

**TABLE 4:** Factors extracted from the exploratory factor analysis (EFA): communalities and factor loadings (*n* = 636).

		**Pattern Matrix**		**Structure Matrix**	
**Item**	**Communality**	**F1**	**F2**	**F1**	**F2**
1	0.583		0.810	0.540	**0.763**
2	0.551		0.833	0.492	**0.737**
3	0.706		0.902	0.586	**0.838**
4	0.521		0.713	0.543	**0.722**
5	0.582		0.713	0.597	**0.762**
6	0.512		0.671	0.558	**0.715**
7	0.567	0.660		**0.749**	0.611
8	0.517	0.406		**0.677**	0.666
9	0.479		0.452	0.623	**0.665**
10	0.575		0.481	0.687	**0.726**
11	0.547		0.420	0.684	**0.696**
12	0.680		0.701	0.679	**0.818**
13	0.607		0.528	0.695	**0.753**
14	0.605	0.713		**0.776**	0.615
15	0.659	0.948		**0.801**	0.509
16	0.633	0.825		**0.795**	0.574
17	0.547	0.606		**0.731**	0.619
18	0.784	0.938		**0.884**	0.626
19	0.701	0.723		**0.831**	0.684
20	0.705	0.781		**0.838**	0.658
Explained variance	–			54.48%	5.83%
Factor correlation	0.745				

*Bolded values highligthed the factor where the item was considered in the Structure Matrix.*

### Confirmatory Factor Analysis (CFA)

#### Two-Factor Model

Mardia’s coefficient for the PSQ-Op was 181.19, indicating violation of the multivariate normality, so a maximum likelihood estimation with bootstrapping was used to generate accurate estimations of standard errors (bias-corrected at the 95% confidence level). The two-factor model derived from EFA was then cross-validated on 1421 participants retained from the entire sample. This solution was run and demonstrated a marginal fit, since the CFI value was above 0.87 and RMSEA and SRMR values were below 0.10 (Bong et al., 2013). The factor loadings of items were above 0.65 (Table 5). The Bollen-Stine value (*p* = 0.001) suggested a poor fit (*p* > 0.05 according to Bollen and Stine, 1992), but this result might have been affected by the large sample size. High correlations between factors were observed.

**TABLE 5:** Confirmatory factor analysis (CFA): fit indexes for each model tested (*n* = 1421).

**Model**	**χ ^2^**	**df**	**Bollen-Stine p**	**CFI**	**RMSEA**	**RMSEA 90% CI**	**SRMR**
Two-factor	2357.96***	169	0.001	0.890	0.096	(0.092–0.099)	0.051
One-factor	3221.37***	170	0.001	0.847	0.112	(0.109–0.116)	0.057
Second-order with correlated errors^*a*^	1551.56***	165	0.001	0.930	0.077	(0.073–0.080)	0.044

***p < 0.001; ^*a*^Added path between error terms for items 3 and 5, 4 and 6, 10 and 11, 15 and 16,; χ^2^-chi-square; CFI, Comparative Fit Index; RMSEA, Root Mean Square Error of Approximation, SRMR, Standardized Root Mean Square Residual.

#### Convergent and Discriminant Validity Evidence

Values of AVE indicated the construct’s convergent evidence. AVE was determined for social issues (AVE = 0.59) and work issues (AVE = 0.54). Concerning the discriminant validity, AVE of the factors was compared to the *r*^2^_DV_. AVE for the two scales was smaller than *r*^2^_DV=__0.76_. These data confirmed that the factors are strongly related to each other, indicating that a unidimensional model or a second-order latent model may be admissible solutions.

#### Unidimensional and Second-Order Models

**FIGURE 6:**
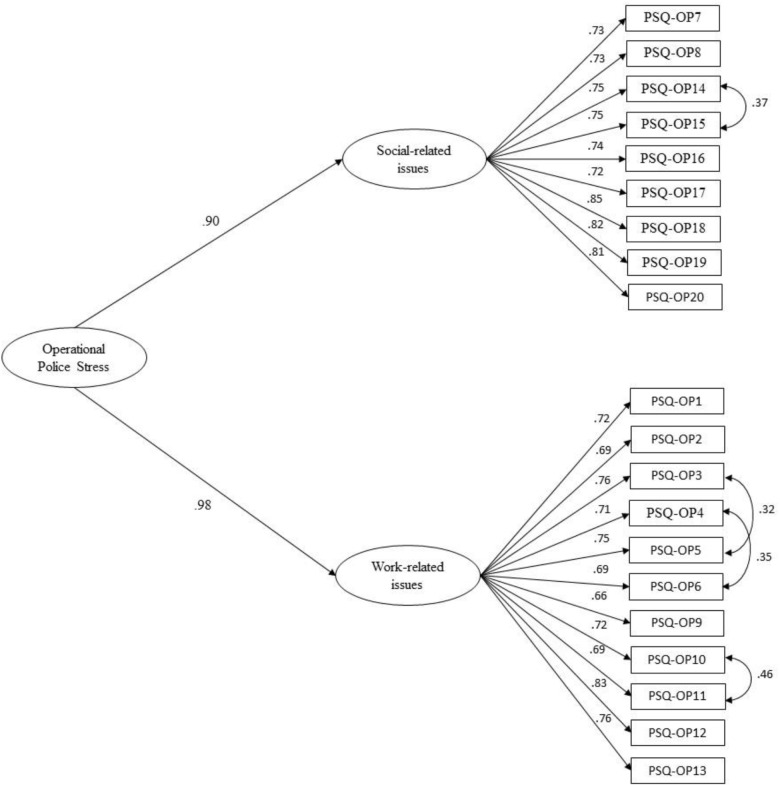
Final confirmatory factor analysis: second-order model with correlated errors.

A single latent model where the factor of operational police stress loads on all 20 items presented a poor fit. Higher error covariance was observed in more than 50% of the items. Based on this result, no additional covariance paths were allowed between error terms. Thus, we examined fit indices for a second-order solution called operational police stress (Table 5), integrating the social and work issues. Based on the high modification indices, allowing errors to covary for items 3 and 5, 4 and 6, 10 and 11, and 15 and 16 improved the model fit. The PSQ-Op second-order construct (Figure 6) presented an acceptable fit based on the values of CFI, RMSEA, and SRMR fit indices. All factor loadings were statistically significant (*p* < 001). The constrained structural weights from operational police stress to social and work factors were high (*Ý* = 0.89, *Ý* = 0.98, *p* < 0.001, respectively).

#### Internal Consistency Evidence

To examine the reliability of the scores in the final model, we used Cronbach’s alpha coefficient and composite reliability. Good internal consistency was obtained in the higher-order construct (α = 0.96) and, simultaneously, for the first-order factors (α = 0.93). Composite reliability coefficients presented values of 0.92 and 0.93 respectively for factors with content related to work and social issues.

### Relationship of PSQ-Op Factors to Distress and Burnout

The PSQ-Op dimensions obtained from the factorial analysis were associated with measures of distress and burnout for the overall sample (Table 6). Positive and moderate to strong correlations (Ratner, 2009) were found, demonstrating the convergent validity of this tool (except for Guilt where correlations were weaker). Higher scores on operational police stress dimensions, such as problems directly related to working conditions and to the impact of work on family and social life, were associated with increased scores in other scales of distress and burnout, except for Enthusiasm, which presented negative correlations. Considering the correlation values, it seems that social-related issues contributes most for burnout and distress compared to work-related issues.

**TABLE 6:** Relationship between PSQ-OP latent variables and distress and burnout symptoms (*n* = 2057).

**Questionnaires’ scales**	**Work-related issues**	**Social-related issues**	**Operational stress**
Work-related issues	1.000		
Social-related issues	0.803**	1.000	
Operational stress	0.953**	0.946**	1.000
Enthusiasm	−0.356**	−0.373**	−0.383**
Psychological exhaustion	0.587**	0.606**	0.628**
Indolence	0.546**	0.578**	0.591**
Guilt	0.131**	0.220**	0.183**
Burnout	0.550**	0.596**	0.602**
Anxiety	0.489**	0.549**	0.545**
Depression	0.497**	0.562**	0.556**
Distress	0.512**	0.577**	0.572**

**p < 0.010.

### Psychological Indicators

Analyses of all the questionnaire scales (Table 7) revealed that the sample presented at least one participant with the minimum or maximum value allowed by the scales’ range. The mean values for operational stress were moderate, being higher for Social-related issues than for Operational stress global score and Work-related issues. Moderate mean values were also found for burnout, being higher for Psychologic Exhaustion and Indolence than for Enthusiasm and Burnout, while Guilt presented a low value. Finally, moderate values were found for Distress, being higher (proportionally) for Anxiety and Distress than for Depression, though very similar. These results are based on mean values of the sample inside each scale range.

**TABLE 7:** Descriptive statistics of operational stress, distress and burnout.

**Scales (range)**	**Minimum**	**Maximum**	**Mean**	**Std. Deviation**
Work-related issues (1–7)	1.00	7.00	4.648	1.496
Social-related issues	1.00	7.00	5.246	1.318
Operational stress	1.00	7.00	4.977	1.328
Enthusiasm (0–4)	0.00	4.00	1.928	0.980
Psychological exhaustion	0.00	4.00	2.379	1.067
Indolence	0.00	4.00	2.231	0.987
Guilt	0.00	4.00	0.858	0.715
Burnout	0.00	4.00	1.877	0.723
Anxiety (4–20)	4	20	9.784	3.938
Depression (6–30)	6	30	14.463	6.160
Distress (10–50)	10	50	24.246	9.743

However, a more detailed analysis was performed considering established levels and cut-off points for each questionnaire (Table 8). For operational stress, the sample presented high stress for 89% on Work-related issues, 84.8% for Operational stress, and 76.2% for Social-related issues, while low stress was recorded at 2.5, 2.7, and 5.9%, respectively for each dimension, suggesting that police officers are experiencing high stress levels and, as referred, not moderates stress according to the mean values. Regarding burnout, 10.6% of the sample presented a very low level and 25.3% a low level for Enthusiasm, while 16.5% presented a high level and 10.6% a critical level for Psychological Exhaustion. For Indolence, those values were respectively 21.8 and 9.7% for Guilt, 20.3 and 8.9%, and for Burnout 21.9 and 10.7%. Finally, for Distress, 21.2% presented low stress, 26.5% high stress, and 28% very high stress, with 54.5% at risk of developing a psychological disorder. This suggests the importance of using cut-off points for each instrument, since they allow us to obtain more detailed information.

**TABLE 8:** Sample’s frequency (and percentage) distribution according established level (cut-off points).

**Questionnaires’ scales**	**Established levels with cut-off points**				
**PSQ-Op**	**Low stress (≤2)**	**Moderate stress (2.1–3.4)**	**High stress (≥3.5)**		
Work-related issues	51(2.5)	175(8.5)	1831(89.0)		
Social-related issues	121(5.9)	368(17.9)	1567(76.2)		
Operational stress	56(2.7)	257(12.5)	1744(84.8)		

**SBI**	**Very low level (*P* ≤ 10%)**	**Low (P11–33)**	**Moderate (P34–66)**	**High (P67–89)**	**Critical level (*P* ≥ 90)**

Enthusiasm	219(10.6)	520(25.3)	701(34.1)	420(20.4)	197(9.6)
Psychological exhaustion	298(14.5)	558(27.1)	643(31.3)	339(16.5)	219(10.6)
Indolence	213 (10.4)	572 (27.8)	624 (30.3)	449 (21.8)	199 (9.7)
Guilt	334 (16.2)	400 (19.4)	722 (35.1)	417 (20.3)	184 (8.9)
Burnout	231 (11.2)	479 (23.3)	676 (32.9)	450 (21.9)	221 (10.7)

**K10**	**Low stress (10–15)**	**Moderate stress (16–21)**	**High stress (22–29)**	**Very high stress (30–50)**	**Risk of mental disorder (22–50)**

Distress	436(21.2)	500(24.3)	545(26.5)	576(28)	1121(54.5)

## Discussion

The literature review showed that the most used psychological measures are not specific nor validated for the specificities of policing tasks. A previous study (Figueiredo-Ferraz et al., 2014) demonstrated that the Spanish Burnout Inventory has adequate psychometric properties for police officers. Furthermore, the Portuguese version of the Police Stress Questionnaire for operational stress also revealed adequate psychometric properties, having a second-order construct but also the possibility to consider two scales that measure work-related issues and social-related issues. However, Irniza et al. (2014) found a unidimensional construct on PSQ-Op for Malay police officers.

Using those two measures combined with a short measure of distress, it was possible to identify burnout, distress, and operational stress among a large national sample of Portuguese police officers, representing nearly 10% of the entire Portuguese police force. The results showed that the mean values of burnout, distress, and operational stress were moderate, but the cut-off points revealed that operational stress and its scales of work-related issues and social-related issues presented high stress levels for more than 75% of the sample (85, 89, and 76%, respectively).

These results are in line with other studies, such as the research by Lipp et al. (2017), who found that 52% of their sample felt stressed, and the study by Brown and Cooper (1996), who also found high stress levels. In the original study for the development of the PSQ-Op, McCreary and Thompson (2006) found that the mean values among Canadian police officers for the 20 items varied between 2.66 and 4.40 and that operational stress had a mean value of 3.32, while the Portuguese sample presented values between 4.02 and 5.57 with operational stress having a mean value of 4.98. Despite the difference between the time of data collection and cultural differences between the countries, given that the maximum value in the range is 7 points, the data suggest a higher level of stress among the Portuguese than the Canadian police officers. Summerlin et al. (2010) found high stress levels among American police officers for some operational tasks (e.g., 68% of the sample considered paperwork to be highly stressful and 73% considered handling the public image to be so), but other tasks were considered as highly stressful only for a few participants (e.g., 16% for activities during days off), while the Portuguese sample considered all tasks as either moderately or highly stressful. Bergman et al. (2016) reported mean values for operational stress among American police officers as 3.4, and 2.91 after a mindfulness intervention. Kaplan et al. (2017) reported mean values for operational stress (also among American police officers) as 3.47. All of these values are smaller than the Portuguese sample in the current study.

The sample presented moderate values for distress symptoms, but 28% of the sample presented very high distress levels, with 55% at risk of developing a psychological disorder. Additionally, the depression scale presented higher values than anxiety. As stated by the European Agency for Safety and Health at Work (EU-OSHA, 2018, 2019a,b), stress has become one of the most important psychosocial risks in the workplace, and it is crucial to develop measures to prevent it. Moreover, anxiety and depression are increasing (OECD, 2019) and are related to distress and burnout, which increases the difficulty of identifying and distinguishing these psychological problems (Bianchi et al., 2015; Golonka et al., 2019; Koutsimani et al., 2019; Bianchi, 2020).

The sample also presented moderate values for burnout, with Guilt having the lowest average, while Psychological Exhaustion and Indolence were higher. However, the analysis of cut-off points revealed that 11% of the sample presented critical values for Burnout, while values between 9 and 11% were found for other burnout dimensions. These values are less than those found by McCarty et al. (2019) who found that 19% of a sample of American police officers suffered with emotional exhaustion and 13% with depersonalization. However, Gutshall et al. (2017) found moderate burnout levels for American police officers, whereas Solana et al. (2013) found high levels of burnout for Spanish police officers. As studies have revealed that burnout decreases self-protective behaviors and increases aggressive behaviors (Euwema et al., 2004; Queirós et al., 2013; Ellrich, 2016), it seems important to assess burnout levels on a regular basis.

Finally, analysis of the correlation between operational stress, distress, and burnout found that higher scores for operational stress, such as problems directly related to working conditions and the impact of work on family and social life, were associated with higher scores for other scales of distress and burnout. Furthermore, it seems that social-related issues interfere most with burnout and distress compared to work-related issues. This may be due to the fact that currently a police officer is not so well respected by society, especially when they are from a national police force that works in urban centers, such as the Portuguese police officers sampled in this study. This means that a large number of participants are away from their families and friends, working in large urban cities such as Lisbon or Oporto, and have difficulties receiving social support from their relatives. This situation contributes to a difficult balance between work and family, and Portugal is a country where professionals work more hours and have more work-family conflicts according to the OECD Better Life Index 2019^10^.

## Conclusion

Burnout and stress among police officers has received increased attention from the scientific community and society, due to the psychological suffering they inflict on the individual, but also because of their impact on the performance of police officers and their interactions with citizens, leading to the increased possibility of all interactions being considered a threat, or to a tendency to use excessive force. Thus, it is crucial to develop stress management interventions (Patterson et al., 2014) and resilience interventions focused on policing specificities, such as those developed by projects like BCOPS (Wirth et al., 2017), HEROES (Thornton et al., 2020), POWER (Papazoglou and Blumberg, 2019), or POLICE (Trombka et al., 2018). However, before implementing an intervention, we need to identify burnout and stress levels, both in the early and later stages of a career. This implies a regular assessment of police officers and will be made easier if short and specific instruments are available and validated for policing stressors. Moreover, occupational health has become a concern, along with the need to identify critical situations early that might, without intervention, lead to situations that are more dangerous. Training mental strength, resilience, or emotional intelligence seems to be a possibility (Papazoglou and Andersen, 2014; Meulen et al., 2017; Romosiou et al., 2018), as well as reflecting the work values of police officers (Basinska and Daderman, 2019), since motivations for becoming a police officer have changed in recent decades (Lester, 1983; White et al., 2010). Furthermore, according to Blumberg et al. (2019, p. 1), new directions should be taken in police academy training, preparing police officers “to meet the contemporary challenges of police work,” and also to develop psychological skills, such as by including in the curricula stress prevention and management programs, as well as topics such as the stress-burnout relationship.

Psychological suffering among police officers can be expressed to others through disengagement or cynical behavior, or impact on the self in the form of depression, sometimes leading to suicide. In fact, suicide among police has become a serious problem and is commonly carried out with the service handgun (Costa et al., 2019). Discussing the current study can help to increase awareness of psychological problems, especially those that are chronic and may result in burnout, and also to reduce burnout stigma and the stigma to seek help (Endriulaitiene et al., 2019). The results highlight the importance of occupational health services in risk prevention and the recovery of workers who play a crucial role in society, such as police officers who deal with safety and security at a national level. Studies that seek to identify police officers’ stress and burnout levels must be continued and will contribute to identifying the risk and protective factors that influence a person’s well-being, quality of life, job performance, and mental health, and also their families and the beneficiaries of police services (society and citizens).

### Theoretical Implications

This study highlights the need to continue research on burnout and stress among police officers to develop our understanding of specific police stressors, such as those evaluated by the PSQ-Op. The literature review reinforces the importance of developing psychological instruments focused on policing tasks, while the data of the sample allow us to verify the relationship between job stress, stress symptoms, and burnout, which present moderate to strong correlations, suggesting they are independent constructs. Furthermore, these results can contribute to scientific research on police forces, a topic that has received increased attention globally, with a particular focus on the causes of stress and burnout. Both the World Health Organization and the European Agency for Safety and Health at Work have highlighted the need to prevent and manage job stress and to valorize mental health in the workplace, as well as the need to view burnout as an occupational phenomenon that must be considered among other psychosocial risks at work.

### Practical Implications

This study provides preliminary data for the Portuguese version of the Police Stress Questionnaire, which presents adequate psychometric properties. Being a short measure, it can be used easily in the future to identify early police officers at risk of developing psychological problems, since occupational stress is related to burnout as an inadequate method of managing chronic job stress. This study used data from a large sample of Portuguese police officers, representing 10% of the entire national force, and the results can be used to identify stress and burnout levels before implementing intervention programs. Additionally, the literature review can be used to identify scientific studies that have assessed stress and burnout among police officers using questionnaires. These kinds of studies can contribute to reducing the stigma of seeking help when police officers confirm that a large number of colleagues are experiencing the same symptoms and difficulties.

### Limitations

In the literature review, the search was focused on studies using questionnaires. This does not reflect all studies of police stress and burnout, which have increased enormously in the last decade. Moreover, the review did not consider post-traumatic stress, which can occur among professionals such as police officers who work in dangerous situations and frequently face critical incidents that can be potentially traumatic. Regarding data collection, the sample came from only one of the Portuguese police forces (called *Polícia de Segurança Pública*, a civil force). Despite the data being a national sample, no data were collected from police officers working in rural areas (from a militarized force called *Guarda Nacional Republicana*), or from a judicial/criminal force (called *Polícia Judicária*), which together comprise the three major Portuguese police forces. Furthermore, data analysis focused on the psychometric properties of the PSQ-Op and on stress/burnout identification levels. The analyses did not compare individual and professional characteristics such as age, gender, or career position. It is worth noting that the meta-analysis of Aguayo et al. (2017) found that sociodemographic factors can be associated with police officers’ burnout.

### Future Research

It will be important in future research to include samples from other Portuguese police forces to verify the invariance of PSQ-Op structure and validity. It will also be necessary to analyze the organizational stressors, which form the second part of the Police Stress Questionnaire. Moreover, the impact of individual and professional characteristics on stress and burnout must be considered, since the literature frequently suggests that different genders deal differently with emotions and stressors, with women feeling more emotional exhaustion, whereas men feel more disengagement, depersonalization, or indolence, and react differently to shift work (Violanti et al., 2018). Additionally, other psychological variables such as coping and resilience must be included, since they can affect stress responses and the process of stress and burnout development (Allison et al., 2019).

## Data Availability Statement

The datasets generated for this study are available on request to the corresponding author, after National Portuguese Police authorization.

## Ethics Statement

This study was carried out in accordance with the recommendations of the Ethics guidelines of the FPCEUP Ethics Committee, having online informed consent from all participants in accordance with the Declaration of Helsinki. The study was approved by the Portuguese National Police.

## Author Contributions

CQ, FP, AP, and CS designed the study. CQ and FP developed the theoretical framework. CQ and AM performed the literature review. AB, AP, and CQ performed the statistical analyses. All authors participated in results’ discussion and final version of the manuscript. All authors of this research manuscript have directly participated in the planning, execution, and analysis of this study.

## Conflict of Interest

The authors declare that the research was conducted in the absence of any commercial or financial relationships that could be construed as a potential conflict of interest.

## Footnotes

1 https://apnews.com/aa2ac2c871a349ecbdb98313b644634b (August 2019).

2 www.facebook.com/zerosuicidiopolicial/ (January 2020) and https://www.elespanol.com/reportajes/grandes-historias/20170909/245476036_0.html (September 2017).

3 https://www.publico.pt/2019/07/17/sociedade/noticia/sindicatos-querem-ministerio-publico-investigue-suicidios-policia-1880178 (July 2019).

4 https://www.euronews.com/2019/10/02/thousands-of-french-police-protest-for-better-working-conditions-and-answer-to-high-suicid (October 2019).

5 https://sicnoticias.pt/pais/2019-11-21-Dia-de-manifestacao-de-policias-em-Lisboa-sob-olema-tolerancia-zero (November 2019).

6 https://www.who.int/mental_health/world-mental-health-day/en/.

7 https://www.who.int/mental_health/evidence/burn-out/en/.

8 https://www.who.int/occupational_health/topics/stressatwp/en/.

9 https://www.midss.org/sites/default/files/psq-op.pdf.

10 https://stats.oecd.org/Index.aspx?DataSetCode=BLI.
